# Hyperactivated RAGE in Comorbidities as a Risk Factor for Severe COVID-19—The Role of RAGE-RAS Crosstalk

**DOI:** 10.3390/biom11060876

**Published:** 2021-06-12

**Authors:** Sara Chiappalupi, Laura Salvadori, Rosario Donato, Francesca Riuzzi, Guglielmo Sorci

**Affiliations:** 1 Department of Medicine and Surgery, University of Perugia, 06132 Perugia, Italy; sarac.chiappalupi@gmail.com (S.C.); francesca.riuzzi@unipg.it (F.R.); 2 Interuniversity Institute of Myology (IIM), 06132 Perugia, Italy; laura.salvadori@uniupo.it; 3 Department of Translational Medicine, University of Piemonte Orientale, 28100 Novara, Italy; 4 Consorzio Interuniversitario Biotecnologie (CIB), 34127 Trieste, Italy; 5 Centro Universitario di Ricerca Sulla Genomica Funzionale (CURGeF), University of Perugia, 06132 Perugia, Italy

**Keywords:** COVID-19 comorbidities, RAGE, *AGER* polymorphisms

## Abstract

The receptor for advanced glycation-end products (RAGE) is a multiligand receptor with a role in inflammatory and pulmonary pathologies. Hyperactivation of RAGE by its ligands has been reported to sustain inflammation and oxidative stress in common comorbidities of severe COVID-19. RAGE is essential to the deleterious effects of the renin–angiotensin system (RAS), which participates in infection and multiorgan injury in COVID-19 patients. Thus, RAGE might be a major player in severe COVID-19, and appears to be a useful therapeutic molecular target in infections by SARS-CoV-2. The role of RAGE gene polymorphisms in predisposing patients to severe COVID-19 is discussed.

## 1. Introduction

Coronavirus disease (COVID)-19, caused by severe acute respiratory syndrome coronavirus (SARS-CoV)-2 [1] (Appendix A), was first detected in December 2019 in Wuhan, China, and has rapidly become a global pandemic due to the high transmissibility and the continuous evolution of the virus, associated with an increasing transmission rate [2]. Over 210 countries worldwide have been involved, with over 145 million infected subjects. People of all ages are susceptible to SARS-CoV-2 infection and experience mild (fever, cough, shortness of breath, muscle aches, loss of taste or smell, diarrhea) or severe symptoms, including pneumonia and acute respiratory distress syndrome (ARDS), with an elevated risk of death due to respiratory failure [3]. About 3.1 million deaths caused by SARS-CoV-2 infection have been registered so far, with up to 96% of dead people showing one or more comorbidities (Table 1).

The comorbidities most frequently associated with severe COVID-19 are chronic lung disease, hypertension, obesity, diabetes, and cardiovascular disease (CVD), typically associated with aging, justifying that aged people are at a higher risk of hospitalization and intensive care unit admission [4]. The presence of comorbidities also dictates the clinical outcomes and length of hospitalization of COVID-19 patients; thus, knowledge of the molecular mechanisms underlying comorbidities and the severe forms of COVID-19 pathology is of extreme importance.

The receptor for advanced glycation-end products (RAGE) is a multiligand receptor of the immunoglobulin superfamily, involved in physiological (e.g., cell proliferation, differentiation, and survival) and pathological (e.g., neurodegeneration, inflammation, and cancer) processes [5]. Interestingly, overexpression and/or hyperstimulation of RAGE characterize COVID-19 comorbidities.

**Table 1:** Deaths in confirmed COVID-19 patients in relation to pre-existing comorbidities.

	Deaths, %		
Comorbidities	Italy	NY	Jakarta
Total ^(1)^	96.1	91.7	31
Hypertension	66.0	53.5	40.4
Diabetes	29.8	34.3	28.6
Coronary artery disease	27.6	13.0	21.1 ^(2)^
Atrial fibrillation	23.1	9.2	*N.S.*
Renal disease	20.2	11.3	9.0
Dementia	19.1	13.7	*N.R.*
COPD	17.1	10.6	5.6
Cancer	16.3	9.2	0.4
Heart failure	15.8	6.6	*N.S.*
Obesity/Hyperlipidemia	10.7	21.6	0.6

The most common comorbidities observed in confirmed COVID-19 deceased patients in Italy (n = 34,142) as of 22 July 2020 [6]; New York State (NY) (n = 41,849) from 1 March 2020 to 26 April 2021 [7]; and Jakarta, Indonesia (n = 4265) from 2 March to 31 July 2020 [8]. (1) Percentages of COVID-19 deceased patients with one or more comorbidities. (2) Reported as cardiac disease. Percentages N.S., Not specified. N.R., Not reported.

Moreover, crosstalk has been reported between RAGE signaling and the renin–angiotensin system (RAS) which is implicated in SARS-CoV-2 infection and disease, and a member of which, namely, angiotensin-converting enzyme 2 (ACE2), is the main cellular receptor of SARS-CoV-2, mediating the entry of the virus into host cells [9]. Following virus binding, ACE2 is internalized, translating into an imbalance of the RAS in favor of proinflammatory members of the system (i.e., angiotensin (Ang) II and the type 1 angiotensin receptor, AT1R) (Appendix A).

Based on several data and considerations, RAGE emerges as an intriguing molecular target in the prevention of severe COVID-19.

## 2. RAGE, a Multiligand Receptor with a Role in Inflammation and Innate Immunity

The recruitment of RAGE by its ligands activates multiple intracellular signaling cascades that depend on the cell type, the ligands involved (including advanced glycation end-products (AGEs), β-amyloid fibrils, high mobility group box-1 (HMGB1), S100 proteins, and nucleic acids), and the RAGE density on the cell surface. Ligand binding causes RAGE oligomerization and association of the RAGE cytoplasmic domain with adaptor proteins (e.g., diaphanous-1 (DIAPH1), TIRAP, and MyD88), which in turn activate intracellular signaling pathways, converging on the transcription factors, NF-κB, AP-1, CREB, STAT3, and/or myogenin, thus regulating the inflammatory response or cell proliferation, survival, differentiation and/or motility [5].

Soluble forms of RAGE (sRAGE), including membrane-cleaved RAGE (cRAGE) and endogenous secretory RAGE (esRAGE), are present in the serum physiologically and act as decoys for the receptor by binding RAGE ligands. cRAGE results from the cleavage of extracellular RAGE by matrix metalloproteinases (MMPs) or ADAM10 (a disintegrin and metalloproteinase domain-containing protein 10) molecules, whereas esRAGE results from a splice variant of RAGE lacking the membrane and cytoplasmic domains [10].

RAGE is a sensor for pathogen-associated molecular patterns (PAMPs), thus behaving as a pattern recognition receptor with a key role in the innate immune response. PAMPs include different pathogen-derived molecules sharing biochemical structures, with lipopolysaccharide (LPS) being the most common. Similar to toll-like receptors (TLRs), the interaction of RAGE with PAMPs activates signaling pathways, leading to NF-κB-driven production of proinflammatory cytokines (e.g., tumor necrosis factor (TNF)-α and interleukin (IL)-1), vasoactive amines, nitric oxide (NO), reactive oxygen species (ROS), and arachidonic acid metabolites, triggering an immune response to eliminate the pathogens or infected cells. Moreover, RAGE binds and mediates the cellular responses of a range of damage-associated molecular patterns (DAMPs, or alarmins, including HMGB1 and S100B) released by cells upon tissue damage or secreted by activated immune cells. In this case, the recruitment of RAGE, together with TLRs (especially TLR4), results in an immune response and sterile inflammation, which are functional to the repair and regeneration of the damaged tissue [5].

RAGE mediates the internalization of complexes formed by released HMGB1 with extracellular DNA, RNA, and other DAMPs or PAMPs, so that they can reach cytosolic proinflammatory receptors, initiating an inflammatory response. On the other hand, disulfide-HMGB1 triggers TLR4, leading to the release of proinflammatory cytokines [11]. The crosstalk between RAGE and TLR4 in response to DAMPs is underlined by the fact that RAGE-positive, TLR4 KO peritoneal macrophages produced almost no proinflammatory cytokines in response to HMGB1 [12], suggesting the existence of a HMGB1/RAGE/TLR4 axis in mediating inflammation in COVID-19. Indeed, HMGB1-RAGE/TLR4 signaling is believed to mediate endothelial activation and dysfunction in COVID-19 conditions [13].

RAGE expression is developmentally regulated, being high until the completion of development, when RAGE is found to be expressed at very low levels or not expressed at all in most tissues. Together with the skin, lung tissue represents an exception, since alveolar epithelial cells (AECs), especially type 1 AECs, express RAGE at high levels physiologically. RAGE is typically found to be re-expressed and overexpressed in conditions of chronic inflammation and in age-related diseases, such as atherosclerosis, Alzheimer’s disease (AD), neuropathy, nephropathy, type 2 diabetes, osteoarthritis, and CVD. RAGE also sustains inflammaging, i.e., the low-grade chronic inflammation that appears during aging and that is at the basis of metabolic disorders associated with chronic morbidity, disability, frailty, and premature death [14].

RAGE is also expressed in several immune cell types, in which it exerts different effects. RAGE plays a crucial role in the differentiation of monocytes and macrophages, whose cellular responses are dependent on the nature of RAGE ligands. RAGE is involved in the adhesion and transmigration of granulocytes during the inflammatory response. Upon activation by AGEs, RAGE increases the phagocytotic activity of neutrophils. Moreover, RAGE is expressed in and promotes the maturation of dendritic cells. In most immune cells, RAGE signaling activates NF-κB, promoting the production of proinflammatory cytokines and the expression of RAGE itself [14].

## 3. Role of RAGE in COVID-19 Comorbidities

People infected by SARS-CoV-2 experience no or mild symptoms in most cases; however, approximately 15% of them develop severe COVID-19, characterized by pneumonia, ARDS, septic shock, and/or multiple organ failure, with elevated mortality [15]. COVID-19 appears in more severe forms and with a higher risk of mortality in people presenting comorbidities such as hypertension, obesity, chronic lung disease, hyperglycemia, diabetes, CVD, and cerebrovascular diseases, especially in elderly people due to the age-related nature of these conditions [4] (Table 1). Notably, COVID-19 is characterized by elevated levels of circulating cytokines, and by a basal state of protein-energy malnutrition and systemic inflammation that are typically associated with aging and metabolic and inflammatory disorders, i.e., COVID-19 comorbidities.

**Figure 1:**
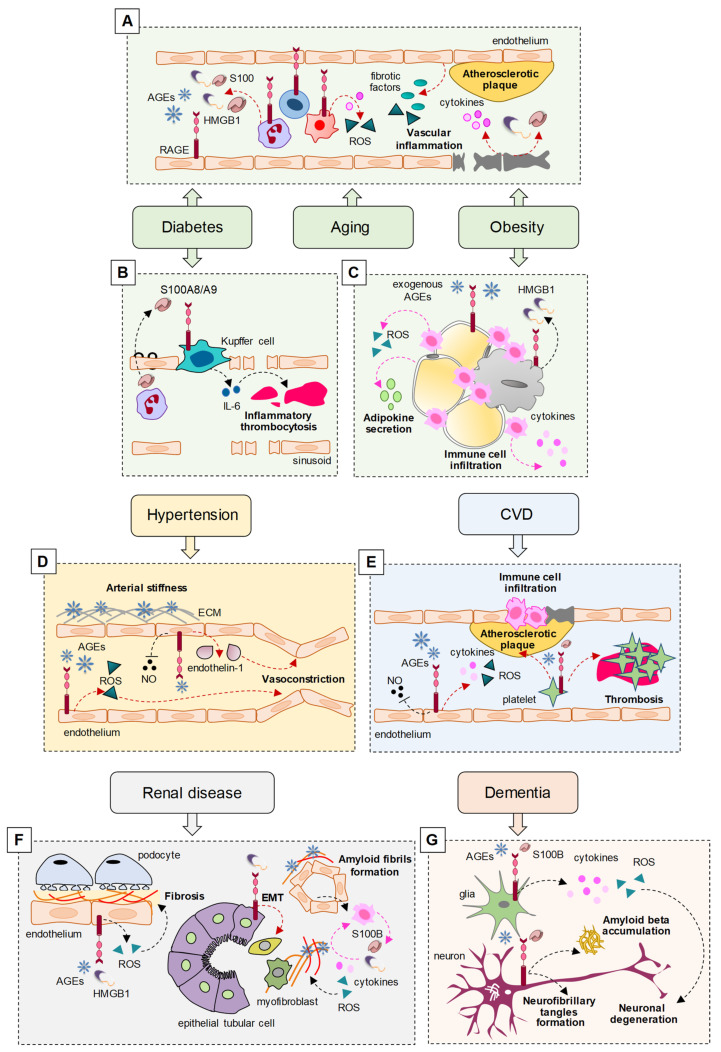
(**A**) In diabetes, obesity, and aging conditions, RAGE, expressed at high levels in activated immune cells and endothelial cells, is overstimulated by elevated serum levels of RAGE ligands (i.e., AGEs, HMGB1, and S100 proteins) leading to oxidative stress (OxS) and the release of cytokines and fibrotic factors, thus predisposing patients to vascular inflammation and atherosclerosis. (**B**) In diabetes, RAGE expressed on hepatic Kupffer cells is stimulated by neutrophil-derived S100A8/A9, resulting in IL-6-dependent inflammatory thrombocytosis. (**C**) In obesity, necrotic adipocytes release HMGB1, which recruits RAGE to induce the secretion of cytokines and adipokines, reactive-oxygen species (ROS) production, and immune cell infiltration. A high-fat diet leads to increased AGE levels in adipose tissue, sustaining RAGE-mediated chronic inflammation and OxS. (**D**) Accumulation of AGEs in the vasculature promotes AGEs/extracellular matrix (ECM) protein cross-linking, thus increasing artery stiffness and promoting hypertension. In addition, AGE–RAGE interaction leads to hypertension by increasing ROS production, by reducing nitric oxide (NO) bioavailability and activity, and by stimulating NF-κB-dependent expression of endothelin-1. (**E**) In cardiovascular disease, elevated serum AGEs interact with endothelial RAGE, leading to ROS and pro-inflammatory cytokine production, and reduced NO levels. AGEs and S100 proteins bind platelet RAGE, favoring platelet aggregation and atherosclerotic plaque formation, predisposing patients to the entry of inflammatory cells into atherosclerotic plaque lesions and increasing the risk of thrombosis. (**F**) In renal disease, increased AGEs and HMGB1 hyperstimulate RAGE, sustaining nephropathy through ROS generation and NF-κB-mediated synthesis of ECM proteins, thus promoting fibrosis. HMGB1 also promotes epithelial–mesenchymal transition (EMT) of tubular cells via RAGE. S100 proteins and HMGB1 released by immune cells induce ROS and inflammation, thus generating additional AGEs that crosslink ECM proteins and induce amyloid fibril formation. (**G**) In the brain, high levels of AGEs and S100 proteins sustain amyloid beta (Aβ) production and accumulation, the formation of neurofibrillary tangles, and neuronal degeneration through RAGE-mediated cytokine and ROS production. CVD, cardiovascular disease.

Interestingly, RAGE has been reported as a major player in sustaining the pathological states in the prevalent comorbidities of COVID-19 (Figure 1) (Table 2). In diabetes, obesity, and aging, RAGE and its ligands are expressed at high levels in activated inflammatory and endothelial cells, sustaining low-grade systemic inflammation and oxidative stress (OxS), which represent risk factors for the development of vascular inflammation and subsequent atherosclerosis, arterial stiffness, and hypertension, predisposing patients to CVD [16,17].

AGEs, the canonical ligands of RAGE, are non-enzymatically glycated proteins or lipids that accumulate in hyperglycemia conditions. The accumulation of endogenous AGEs also occurs in the presence of high OxS, resulting in sugar oxidation, which facilitates protein glycation, and during inflammatory processes, in which AGE formation is favored by the myeloperoxidase secreted by activated neutrophils. Since hyperglycemia, OxS, and inflammation typically occur during aging, it is not surprising that the accumulation of AGEs is a common hallmark in elderly people and that these subjects are predisposed to RAGE hyperactivation [16]. An additional and major source of (exogenous) AGEs is represented by processed, baked, and roasted foods, typical of the modern western diet [18], which deserves particular attention in consideration of the predisposing role of AGEs to COVID-19 comorbidities.

**Table 2:** RAGE ligands and RAGE expression and activity in COVID-19 comorbidities.

Comorbidities	RAGE Ligands	RAGE Expression	RAGE Activity	Refs
Hypertension	High serum AGEs, S100B, and HMGB1.	High expression of RAGE; low serum sRAGE and esRAGE.	AGE-RAGE generates pro-inflammatory cytokines, vascular adhesion molecules, and ROS in endothelial cells; AGEs reduced NO and increased endothelin-1.	[19,20,21,22,23,24]
Diabetes	High serum AGEs, S100B, and HMGB1.	Increased expression of RAGE in monocytes. Low serum sRAGE and esRAGE in type 2 diabetes; high serum sRAGE in critically ill diabetic patients.	AGE-RAGE results in atherosclerosis through activation of NF-κB and NADPH oxidase, leading to increased expression of proinflammatory cytokines, pro-fibrotic factors, and ROS. S100 proteins and HMGB1 promote the release of proinflammatory cytokines and ROS formation via RAGE, contributing to atherogenesis.	[16,17,25,26,27,28]
Coronary artery disease	High serum AGEs, S100A6, S100A12, S100B, S100P, and HMGB1.	High expression of RAGE in atherosclerotic plaques and coronary arteries. High serum sRAGE; low serum esRAGE.	RAGE signaling induces inflammation and OxS, leading to amplification of the atherosclerotic inflammatory response.	[29,30,31,32,33,34,35,36,37]
Atrial fibrillation	High serum AGEs, and HMGB1.	High expression of RAGE. High serum sRAGE and esRAGE.	AGE-RAGE induces atrial fibrosis, inflammation, and OxS. HMGB1-RAGE promotes platelet aggregation, activation of coagulation factors, and fibrin formation.	[38,39]
Renal disease	High serum AGEs and HMGB1.	Low serum esRAGE in patients with end-stage renal disease.	AGE-RAGE induces inflammation and OxS in podocytes. Immune cells recruited to the nephrons release S100 proteins and HMGB1, inducing OxS and inflammation, and generating additional AGEs; AGEs crosslink local ECM proteins and induce amyloid fibril formation.	[40,41,42,43,44,45]
Dementia	High levels of AGEs in neurons and vessels in vascular dementia. High levels of AGEs, S100A9, S100A12, and S100B in AD brains	High expression of RAGE. High serum sRAGE and esRAGE in patients with vascular dementia; low serum sRAGE in AD patients.	RAGE participates in Aβ production and accumulation in AD. Interaction of Aβ with RAGE induces inflammation and OxS, exacerbating Aβ deposition.	[5,46,47,48,49,50,51,52]
COPD	High levels of AGEs, HMGB1, and S100B.	High expression of RAGE in lungs. Low levels of sRAGE in serum and BAL.	RAGE activation recruits neutrophils to airway space. Degradation of sRAGE by neutrophil-derived proteases leads to hyperactivation of RAGE, and persistence of neutrophil recruitment and inflammation.	[53,54,55]
Heart failure	High serum pentosidine and HMGB1.	High RAGE expression in the heart. High serum sRAGE and cRAGE; low serum esRAGE.	RAGE signaling sustains cytokine production, inflammation, OxS, and fibrous tissue deposition	[56,57,58]
Obesity/Hyperlipidemia	High serum AGEs, S100A4, S100A8/A9, S100B, and HMGB1.	High RAGE expression in adipose tissue. Low serum level of sRAGE and esRAGE.	HMGB1 released by necrotic adipocytes interacts with RAGE, inducing pro-inflammatory cytokines and ROS; S100A4, S100A8/A9, and S100B induce inflammation, interacting with RAGE; AGE accumulation in adipose tissue may contribute to obesity-associated insulin resistance.	[59,60,61,62,63,64]

### 3.1. Diabetes and Obesity

In diabetic patients, the increase in plasma levels of AGEs is responsible for a non-physiological hyperactivation of RAGE, leading to atherosclerosis through the activation of NF-κB and NADPH oxidase, resulting in the increased expression of proinflammatory cytokines and pro-fibrotic growth factors, and the generation of ROS, which is causative of severe complications, including CVD and nephropathy [16,17].

Together with AGEs, the serum levels of other RAGE ligands, such as HMGB1 and S100 proteins, are elevated in diabetic and obese patients and, similarly to AGEs, their increase has been associated with endothelial dysfunction and cardiovascular complications in diabetic and non-diabetic subjects [17]. In diabetic conditions, AGEs induced by hyperglycemia lead to the release of HMGB1, which plays a crucial role in the pathogenesis of diabetic complications, promoting the release of proinflammatory cytokines and ROS formation, also via the interaction with RAGE [25]. In response to hyperglycemia, neutrophil-derived S100A8/A9 interacts with RAGE on hepatic Kupffer cells, resulting in increased production of IL-6, a cytokine implicated in inflammatory thrombocytosis. The inhibition of S100A8/A9 reduces diabetes-induced thrombocytosis and decreases atherogenesis in diabetic mice [26].

The levels of AGEs increase in liver and adipose tissue following a high-fat diet, contributing to obesity and promoting the secretion of proinflammatory cytokines (adipokines) from adipose tissue mainly through their interaction with RAGE, sustaining low-grade chronic inflammation and OxS, leading to diabetes, insulin resistance, and CVD [18]. Mice lacking RAGE fed with high-fat diets showed reduced weight gain, reduced macrophage infiltration in the adipose tissue, and reduced insulin resistance, demonstrating that RAGE is a major contributor in the development of obesity and adipose tissue-related inflammation [59].

In obesity conditions, HMGB1 released by necrotic adipocytes interacts with RAGE, inducing the secretion of proinflammatory cytokines, and recruits immune cells that in turn induce additional adipocyte death through the further release of proinflammatory cytokines and ROS production [60]. The S100 protein family members, S100A4, S100A8/A9 heterodimer, and S100B have all been implicated in the pathophysiology of obesity-associated inflammation via their interaction with RAGE [61].

Notably, diabetes and obesity are associated with increased susceptibility to infections. COVID-19 patients with diabetes mellitus or obesity are at a higher risk of worse outcomes (including respiratory and multiple organ failure) and higher mortality. In diabetic patients, reduced mobilization of polymorphonuclear leukocytes, chemotaxis, and phagocytic activity, and inhibition of TNF-α have been reported, resulting in a compromised innate immune response against SARS-CoV-2, leading to excessive and uncontrolled inflammation and a hypercoagulable state [65]. Interestingly, diabetic mice exhibited an excess of proinflammatory cytokines in response to infections with Gram-negative bacteria in comparison with non-diabetic mice, driving lethal hyperinflammation through TLR4 and RAGE via their common adaptor protein, MyD88. Inhibition of RAGE with FPS-ZM1 protected diabetic mice against bacterial infection [66].

Similarly to diabetic patients, obese patients show a defective immune response, endothelial dysfunction, hypercoagulability, and thrombosis following SARS-CoV-2 infection. Obesity is particularly detrimental in patients with COVID-19 likely because of the high expression level of ACE2 in the adipose tissue (several-fold higher than that in the lungs), suggesting that adipose tissue is more vulnerable to SARS-CoV-2 infection, representing a reservoir of the virus, and driving multiorgan damage. This condition is exacerbated by the production by adipocytes of angiotensinogen, which is converted by renin and ACE into proinflammatory Ang II [67].

Diet-induced obese mice infected with an influenza A virus (IAV) showed a significant increase in the mortality rate, a marked delay in antiviral and proinflammatory cytokine production, and a substantial reduction in natural killer (NK) cell cytotoxicity in the lungs compared to lean control mice. Moreover, in infected obese mice there was a more severe lung pathology, characterized by a late and heightened inflammatory response, likely due to increased numbers of infected lung cells, infiltration of cytokine-producing T cells, and reduced production of antiinflammatory cytokines [68].

### 3.2. Hypertension

The AGE-RAGE axis is involved in arterial stiffness and hypertension. In patients with hypertension, the plasma levels of AGEs are positively correlated, and serum levels of sRAGE and esRAGE inversely correlate with arterial stiffness and hypertension [19]. AGEs can induce hypertension by both altering the arterial compliance/stiffness and interacting with membrane RAGE on the cell surface, resulting in changes in cell function. Cross-linking between AGEs and proteins of the extracellular matrix (ECM), such as collagen and elastin, increases the artery stiffness [20]. AGE-RAGE interaction leads to increased ROS production, which in turn is responsible for the increase in the total peripheral vascular resistance, suggesting that AGEs, through the production of ROS, may induce hypertension irrespective of arterial stiffness [19]. Moreover, AGEs may induce hypertension by reducing the bioavailability and activity of the vasodilator molecule, NO [21], and increasing the expression of the vasoconstrictor factor, endothelin-1 through NF-κB [22].

Of note, antihypertensive pharmacological treatments were shown to have a beneficial role in elderly COVID-19 patients with comorbid hypertension since the clinical outcomes were significantly improved in the case of treatment with angiotensin receptor blockers (ARBs), ACE inhibitors, beta-blockers, or calcium channel blockers (CCBs) compared with patients who took no drugs [69]. Nevertheless, different antihypertensive pharmacological treatments were associated with different protection against severe COVID-19, as a large nationwide retrospective cohort study showed that antihypertensive people exposed to ACE inhibitors or ARBs were at less risk of hospitalization with COVID-19 and less risk of intubation or death compared with individuals exposed to CCBs [70], pointing to a crucial role of RAS in COVID-19 pathology (see Section 4).

### 3.3. Cardiovascular Disease

Coronary artery disease (CAD) represents the main form of CVD and is due to atherosclerosis. The main risk factors are represented by dyslipidemia, diabetes, hypertension, cigarette smoking, and obesity, all characterized by elevated RAGE expression and signaling. Indeed, AGE/RAGE signaling has an atherogenic role, whereas sRAGE has antiatherogenic effects. A high AGE/sRAGE ratio (also known as AGE-RAGE stress) results in the development and progression of CAD [29]. However, conflicting results have been reported about the serum levels of sRAGE in CVD patients, with lower or higher levels of sRAGE found, depending on the type of patients investigated. sRAGE was found to be elevated in type 1 or type 2 diabetes and end-stage renal disease [30,31]; in contrast, the serum levels of esRAGE were lower in CAD patients, and low levels of esRAGE were associated with increased mortality [32]. Other RAGE ligands have been implicated in CVD. Subjects with acute coronary syndrome showed increased serum levels of S100A6, S100A12, S100B, and S100P in comparison with control groups and healthy people [33,34]. Increased serum levels of HMGB1 are associated with CAD in type 2 diabetic patients and nondiabetic people [35].

As in the case of hypertension, the molecular mechanisms at the basis of RAGE-mediated effects in CVD include the production of OxS, pro-inflammatory cytokines, and vascular adhesion molecules, and the impairment of NO production by inhibiting the expression of NO synthase in the endothelium. AGEs and S100 proteins bind RAGE on the surface of platelets, upregulating adhesion molecules and glycoproteins and favoring platelet aggregation and atherosclerotic plaque formation, increasing the risk of microthrombus [36]. Following the recruitment of RAGE, inflammatory cells enter the atherosclerotic plaque lesions through the damaged endothelial barrier, contributing to the development of atherosclerosis [37].

The AGE–RAGE axis is also involved in atrial fibrillation by inducing atrial fibrosis, inflammation, and OxS, translating into atrial electrical remodeling [38]. HMGB1-RAGE promotes thrombosis in patients with atrial fibrillation, contributing to platelet aggregation, activation of coagulation factors, and fibrin formation [39].

RAGE and RAGE ligands are upregulated in injured heart, and the serum levels of RAGE ligands and sRAGE correlate with the degree of heart failure [56]. Interestingly, the serum concentration of the AGE pentosidine was found to be an independent risk factor for heart failure, since serum pentosidine positively correlated with the risk of cardiac events [57]. Increased serum HMGB1 and cRAGE, and decreased esRAGE levels, were found in heart failure patients, correlating with the severity of the pathology in diabetic and non-diabetic patients [58].

### 3.4. Lung Disease

RAGE is involved in non-infective and infective pulmonary diseases. Indeed, increased amounts of RAGE and/or RAGE ligands have been reported in pulmonary fibrosis, acute lung injury (ALI), ARDS, pneumonia, and cystic fibrosis (CF), and were demonstrated to sustain the pathological outcomes in these conditions [71]. Moreover, serum sRAGE is a recognized marker of lung epithelial injury, associated with prognostic and pathogenic values in patients with ARDS [72]. In an experimental model of LPS-induced ALI the use of an anti-RAGE blocking antibody reduced the upregulation of RAGE expression and NF-κB activation in the lung, and consequently restrained inflammatory lung injury [73].

Due to the amount of AGE precursors (including glycotoxins) contained in cigarette smoke, RAGE expressed by AECs is highly recruited in smokers, predisposing them to chronic obstructive pulmonary disease (COPD), ultimately resulting in emphysema. HMGB1 was found to be increased in the lungs of smokers with COPD compared to non-COPD smokers or never-smokers, and a positive correlation between the serum levels of S100B and the severity of pathology was reported in a cohort of COPD patients [53].

DAMPs released upon lung epithelial damage activate RAGE, thus sustaining the recruitment of neutrophils to sites of inflammation in the airway space of COPD patients. By upregulating the RAGE ligand, Mac-1/CD11b on their surface, activated neutrophils interact with AECs via a RAGE-dependent mechanism, thus amplifying the inflammatory response [54].

Reduced levels of serum sRAGE have been observed in COPD patients compared with healthy subjects [55], and a correlation between a lack of sRAGE in bronchoalveolar lavage and high levels of airway neutrophils has been observed in asthmatic and COPD patients. This is likely due to the degradation of sRAGE by neutrophil-derived proteases, leading to the hyperactivation of RAGE, with the persistence of neutrophil recruitment and inflammation [54].

RAGE signaling is detrimental during IAV-induced pneumonia, which was found to be associated with the enhanced expression of this receptor, together with HMGB1, in lungs. RAGE-ablated mice infected with IAV showed enhanced immune responses and better outcomes compared with IAV-infected wild-type mice [74]. Moreover, together with TLR4 and TLR9, RAGE recruited by HMGB1 promotes human adenovirus type 7 (HAdV-7) replication and signaling in a severe form of pediatric pneumonia [75].

### 3.5. Renal Disease

AGEs/RAGE signaling is directly involved in the pathogenesis and progression of diabetic nephropathy due to the generation of OxS and activation of NF-κB, increased synthesis of ECM proteins, and subsequent perturbation of podocytes and tubular cell homeostasis [40]. Elevated levels of HMGB1 were found in patients with chronic kidney diseases, and HMGB1 has been detected in urine, blood, and cell types of renal parenchyma in renal disease conditions [41]. HMGB1 enhances the epithelial–mesenchymal transition of tubular cells via RAGE recruitment, thus promoting renal fibrosis in diabetic nephropathy [42].

In renal diseases, immune cells recruited to the nephrons release S100 proteins and HMGB1, inducing ROS and inflammation, thus generating additional AGEs that crosslink local ECM proteins and induce amyloid fibril formation [43]. RAGE^−/−^ mice fed with a Nε-(carboxymethyl)-lysine (CML)-enriched diet for 18 months showed the accumulation of CML in the renal tubules but were found to be protected against nephrosclerosis lesions (i.e., hyalinosis, tubular atrophy, fibrosis, and glomerular sclerosis) and renal senile amyloidosis, and expressed less inflammatory and fibrotic mediators compared with control mice [44].

### 3.6. Dementia

A significantly increased risk for COVID-19 and an association with severe outcomes of the pathology have been observed in patients with dementia, including AD [76,77]. On the other hand, neuronal injury and glial activation have been reported in patients with moderate to severe COVID-19 with or without dementia [78]. RAGE signaling is likely to have a role in these conditions, since (i) a correlation between the levels of the most abundant AGE, CML, in neurons and vessels and cognitive impairment has been reported in subjects with vascular dementia [46]; (ii) toxic AGEs have been found in the neurofibrillary tangles and neurons of the hippocampus and parahippocampal gyrus of AD patients [47,48]; and (iii) S100B, S100A9, and S100A12 levels are increased in AD brains [49]. It is known that RAGE plays a critical role in AD, participating in amyloid beta (Aβ) production and accumulation, the formation of neurofibrillary tangles, failure of synaptic transmission, and neuronal degeneration by acting as an inflammatory intermediate and a critical inducer of OxS [5,50].

### 3.7. RAGE in Severe COVID-19

Thus, in subjects with comorbidities, the systemic accumulation of RAGE ligands might predispose to severe pulmonary pathology and multiorgan damage following coronavirus infection. In the presence of elevated RAGE ligands, the hyperactivation of RAGE occurs, leading to a basal sub-clinical inflammatory state that primes the lung for an excessive and unsuccessful response to the virus.

The potential key role of RAGE in the severe forms of COVID-19 is highlighted by very recent findings about some of its ligands: (i) serum levels of AGEs were found to be higher in COVID-19 patients with lung involvement than in asymptomatic patients [79]; (ii) S100A12 was found to be one of the differentially expressed proteins in the bronchoalveolar lavage fluid of critical COVID-19 patients [80]; (iii) elevated levels of S100B and S100A8/A9 were detected in the serum of COVID-19 patients, significantly correlating with the severity of disease [81,82]; and (iv) the serum levels of S100A8/A9 and HMGB1 at hospital admission were found to be increased in intensive care unit (ICU) compared to non-ICU patients, and in fatal outcomes compared to surviving patients, in a retrospective study of COVID-19 patients [83]. Altogether, these data indicate that S100A12 is involved in COVID-19 lung tissue damage, and that the elevation of AGEs S100B, S100A8/A9, and HMGB1 in the serum of SARS-CoV-2-infected people is associated with worse outcomes and increased mortality, pointing to an overall convergence of RAGE signaling in severe COVID-19.

Notably, in a meta-analysis of the transcriptomic response of infected human cells, comparative gene set enrichment analysis showed that AGE/RAGE was one of the twenty commonly activated pathways upon SARS-CoV, middle east respiratory syndrome coronavirus (MERS-CoV), and SARS-CoV-2 infections, confirming that RAGE signaling is crucially involved in coronavirus pathogenesis [84].

Excess inflammation and OxS contribute to the generation of the cytokine storm in COVID-19 patients, resulting in endothelial dysfunction, which is causative of multiorgan damage and more severe illness. Endothelial dysfunction is a common clinical feature of coronavirus infections, in which the endothelium undergoes disruption due to increased proinflammatory mediators and subsequent deregulation of the coagulation cascade [85]. The HMGB1/RAGE axis plays a major role in this process. HMGB1 is passively released by injured endothelial cells and, behaving as a DAMP, induces the expression of proinflammatory cytokines, chemokines, adhesion molecules (ICAM-1 and VCAM-1), and RAGE itself. This way, HMGB1 induces the recruitment of macrophages, which upregulate RAGE and the activity of which translates into the further release of HMGB1 and cytokines propagating the inflammatory response, representing the early stage of atherosclerosis and predisposing to acute ischemic stroke [16], a condition commonly observed in severe COVID-19 patients [85]. Finally, RAGE has been involved in the formation of neutrophil extracellular traps (NETs, i.e., extracellular webs made of DNA, histones, microbicidal proteins, and oxidant enzymes) [17] that are released by neutrophils to restrain SARS-CoV-2 infection, and the excess formation of which causes respiratory failure and thrombosis in severe COVID-19 [86]. DAMPs, and in particular HMGB1, are potent activators of NETosis [87]. Platelet-derived disulfide HMGB1 promotes NET formation in a RAGE-mediated manner, leading to the exposure of additional HMGB1 on NETs’ extracellular DNA strands, thus leading to a vicious circle of coagulation and inflammation, predisposing the subject to thrombosis [88]. In addition, neutrophils and platelets release S100 proteins, which facilitate thrombus formation through RAGE activation [89].

## 4. RAGE and the Renin–Angiotensin System. Overlapping Pathways and Biased Signaling with Potential Relevance in COVID-19

The RAS (Appendix B), an endocrine system with pleiotropic activities, mainly known for its involvement in the control of blood pressure, has a prominent role in COVID-19 pathology, starting from the entry of the virus into cells through the interaction of the viral protein, spike, with the receptor, angiotensin-converting enzyme 2 (ACE2). Following interaction with SARS-CoV-2, virus-dependent ACE2 downregulation is responsible for the accumulation of ACE1-derived Ang II, which mediates the inflammatory response and parenchymal injury in lungs and other organs by interacting with AT1R and activating NF-κB. ACE2 glycosylation, as occurring under hyperglycemia conditions typical of diabetes, increases the binding affinity of ACE2 to the virus and favors the spreading of the virus to multiple organs [90]. Preventing imbalances in RAS members or favoring the activity of Mas receptor (MasR) or AT2R is a therapeutic strategy to restrain SARS-CoV-2-dependent tissue damage in COVID-19 patients [9].

Crosstalk between RAGE signaling and the RAS cascade has been reported in several cell types. In endothelial cells in conditions of hyperpermeability (as occurs in COVID-19 comorbidities), a loop sequence of events takes place, fuelling inflammation and OxS. Ang II interacts with AT1R, activating NF-κB and leading to the expression and release of HMGB1, along with the expression of AT1R itself and RAGE. By interacting with RAGE, extracellular HMGB1 activates additional NF-κB to further increase HMGB1, AT1R, and RAGE expression. The use of sRAGE hampers the Ang II-induced expression of AT1R and RAGE in these conditions [91]. Another example is offered by podocytes, in which treatment with AGEs results in increased expression of angiotensinogen, Ang II, and AT1R via phosphoinositide 3-kinase and in podocyte apoptosis [92]. A role of RAGE in sustaining Ang II-induced proinflammatory signaling has also been reported in macrophages and splenocytes [93]. Conversely, Ang II controls HMGB1 expression and release by macrophages. Indeed, Ang II induces the polarization of proinflammatory M1 macrophages through the upregulation, acetylation, and release of HMGB1, which in turn upregulates Ang II, thus activating a positive loop [94].

A link between RAGE and AT1R signaling has been demonstrated in the pathogenesis of diabetic atherosclerosis. In the early stages of diabetes, increased AT1R staining is associated with the expression of RAGE and its ligand, EN-RAGE (S100A12), in aortic vascular smooth muscle cells (VSMCs), and treatment of VSMCs with Ang II increases RAGE expression via AT1R. Moreover, AT1R deficiency or treatment with ARBs prevented the diabetes-induced hyperactivation of the ligand/RAGE axis and monocyte chemoattractant protein (MCP)-1-related inflammation, and atherogenesis in diabetic mice [95]. On the other hand, mice genetically lacking the RAGE gene (*Ager*) and susceptible to atherosclerosis (*Ager*/*Apoe*-double-KO mice) were found to be protected against Ang II-induced plaque accumulation across the aortic arch [93]. Treatment with the ACE inhibitor (ACEi), benazepril significantly suppressed the accumulation of AGEs and RAGE expression, thus reducing NADPH oxidase upregulation, ROS generation, NF-κB activation, and VCAM-1 and TGF-β1 levels in the kidneys of spontaneously hypertensive rats [96].

**Figure 2:**
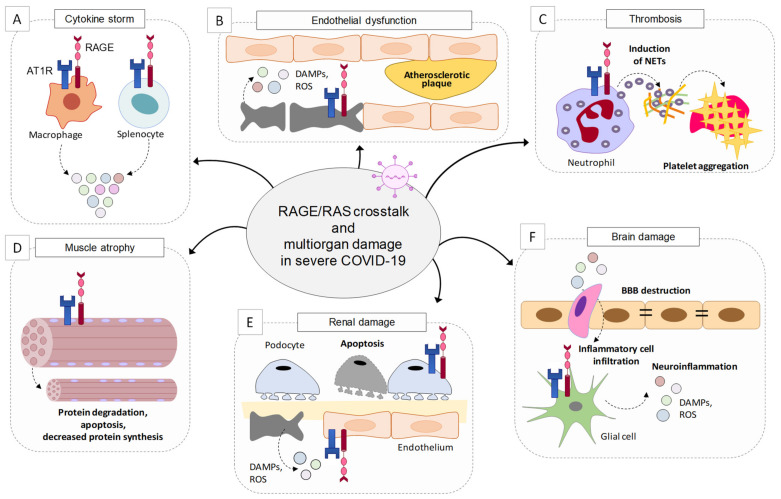
RAGE/RAS crosstalk might take place in several tissues sustaining multiorgan damage in severe COVID-19 conditions. RAGE/RAS signaling might (**A**) sustain cytokine storm in macrophages and splenocytes; (**B**) induce endothelial dysfunction by increasing capillary permeability, the release of damage-associated molecular pattern molecules (DAMPs), and reactive-oxygen species (ROS) production, leading to atherosclerotic plaque formation; (**C**) predispose to thrombosis by inducing the formation and release of neutrophil extracellular traps (NETs) by neutrophils and subsequent platelet aggregation; (**D**) induce muscle atrophy by stimulating apoptosis, increasing protein degradation, and reducing protein synthesis; (**E**) sustain renal damage by inducing podocyte apoptosis and endothelial dysfunction; and (**F**) induce brain damage by altering the blood-brain barrier (BBB) favoring the entry of immune cells, and neuroinflammation.

The existence of an interplay between RAGE and AT1R signaling is stressed by the fact that the formation of RAGE/AT1R heteromeric complexes at the plasma membrane has been reported at the basis of the Ang II/AT1R-dependent transactivation of RAGE, leading to inflammation and atherogenesis, independently of the presence of RAGE ligands [93]. These results point to a role of RAGE/AT1R crosstalk in severe COVID-19 and COVID-19 comorbidities (Figure 2).

The intracellular signaling activated by the recruitment of RAGE or members of the RAS may be affected by the absence or presence of specific co-receptors or co-factors. Indeed, it is known that some ligand-receptor-effector complexes may generate distinct conformations, leading to the preferential activation of certain intracellular signaling pathways rather than others, a phenomenon known as “biased signaling” [97]. The extensive literature reporting crosstalk between RAGE and the RAS cascade suggests that a biased signaling system is established in several pathological settings in which RAGE overstates the deleterious effects of the RAS, especially when RAGE is overexpressed or overstimulated by an excess of its ligands. Consistent with the interaction between RAGE and RAS signaling, ARBs or ACEi reduce the proinflammatory action of RAGE in several experimental settings of diseases: (i) the use of the ARBs, telmisartan, irbesartan, and candesartan, which proved to be efficacious in the prevention and acute treatment of stroke, reduces RAGE expression, thus inhibiting the HMGB1/RAGE axis in stroke conditions [98]; (ii) telmisartan blocks the upregulation of RAGE expression and the release of sRAGE induced by treatment with Ang II in cultured endothelial cells [99]; (iii) losartan reduces the HMGB1 release in sepsis conditions [100]; (iv) candesartan reduces AGE accumulation and subsequent albuminuria by attenuating RAGE expression and downregulating NADPH oxidase and iNOS expression in kidneys of type 2 diabetic mice [101]; (v) the ACEi ramipril reduces the accumulation of AGEs and inhibits the activation of MMP-2 and ROS generation induced by the AGE/RAGE axis in experimental models of diabetic nephropathy [102]; and (vi) treatment with ACEi resulted in a significant increase in sRAGE and the reduction of AGE formation in bovine aortic endothelial cells, diabetic rat models, and type 1 diabetes patients, and in a decrease of renal full-length RAGE in ACEi-treated rats [103].

RAGE/RAS crosstalk might be involved in the abnormal activation of platelets and the release of NETs. Spike protein/ACE2 interaction directly activates platelets, leading to the release of HMGB1, together with coagulation factors and inflammatory cytokines, and potentiating platelet prothrombotic function [104]. RAGE/RAS-dependent ROS production and excessive activation of NF-κB might exacerbate neutrophil infiltration and the induction of NETs, which in turn collaborate with platelet RAGE to induce platelet aggregation [105]. The role of RAGE/RAS in thrombosis also deserves attention in consideration of the undesired effects reported for adeno-associated virus (AAV)-based vaccines [106].

Due to its established role in neuroinflammation [5,50], RAGE signaling might be critical in exacerbating the neurological damage and outcomes of COVID-19 patients through its interaction with the RAS. ACE2 is expressed by neurons and glial cells, including astrocytes and microglia, making the nervous system a potential target of SARS-COV-2. The increase in circulating levels of chemokines and interleukins subsequent to SARS-COV-2 pulmonary infection compromises the blood–brain barrier (BBB), favoring the entry of the virus into the brain parenchyma and leading to inflammation, with the subsequent risk of neurological damage [107].

The deleterious crosstalk between RAGE and RAS might be active also in the muscles of severe COVID-19 patients who develop cachexia [108], a complex metabolic syndrome characterized by the loss of muscle mass and body weight, since both RAGE and RAS signals play crucial roles in inducing muscle protein breakdown [109]. Skeletal muscle tissue expresses a considerable amount of ACE2 [110], making muscles potential targets of SARS-CoV-2. Deletion of ACE2 in mice translated into early manifestations of muscle weakness with signatures of muscle senescence [111], suggesting that the SARS-CoV-2-dependent downregulation of ACE2 might predispose COVID-19 patients to cachexia. At the muscle level, Ang II induces protein degradation and apoptosis, and decreases protein synthesis, and these detrimental effects are partly mediated by AT1R [112]. Increased serum levels of Ang II have been found in cachectic patients [113]. RAGE is highly re-expressed in adult skeletal muscle in wasting conditions occurring in response to aging, genetic disorders, inflammation, cancer, and metabolic alterations, and the serum levels of several RAGE ligands are increased in cachexia, thus sustaining RAGE activity [10,109]. Exogenous administration of the ACE2 cleavage product Ang-(1-7) or deletion of RAGE rescued muscle wasting in age-related and cachexia conditions, respectively [109,111]. The reported increased expression of ACE2 in an experimental model of Duchenne muscular dystrophy (DMD) [114], in which RAGE is also expressed and chronically stimulated [115], points to a susceptibility of DMD muscles to SARS-CoV-2 infection, and the vulnerability of DMD patients to accelerated muscle wasting when affected by COVID-19.

All comorbidities associated with severe COVID-19 are characterized by sympathetic overactivation, exacerbating the deleterious effects of the pathologies on the affected organs. In COVID-19 patients, the enrollment of the sympathetic system is further sustained by the increased induction and release of Ang II, thus establishing a vicious circle, worsening the pathological outcomes [116]. Due to the crosstalk between RAGE and the RAS, it is likely that RAGE also has a role in overactivating the sympathetic system in severe COVID-19 conditions.

**Figure 3:**
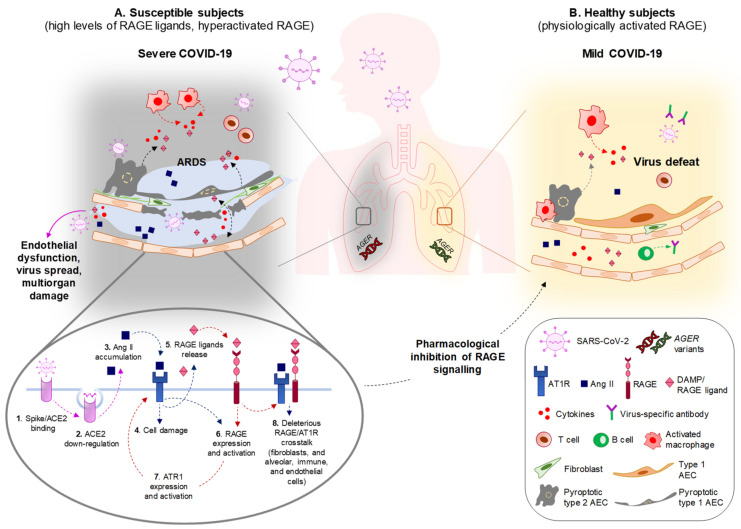
(**A**) In susceptible subjects (i.e., aged subjects and patients with preexisting comorbidities) a basal state of lung inflammation and high levels of circulating RAGE ligands occur, predisposing them to severe COVID-19. Upon recognition and binding of the viral spike protein with ACE2 receptor (1), SARS-CoV-2 enters type 2 alveolar epithelial cells (AEC) and leads to downregulation of ACE2 (2), resulting in the unopposed accumulation of Ang II (3), which mediates the inflammatory response and parenchymal injury in the lungs (4). Infected AECs undergo pyroptosis and release DAMPs, including RAGE ligands (5), leading to excess RAGE expression and activation (6). In these conditions, RAGE activity increases AT1R expression (7), and might transactivate AT1R, reinforcing the deleterious effects of Ang II/AT1R in alveolar epithelial, immune, and endothelial cells, and in fibroblasts (8). Ang II/AT1R induce the further release of RAGE ligands (5), thus establishing a detrimental loop between AT1R and RAGE signaling. The result is increased capillary permeability, interstitial edema, fibrosis, further cytokine-dependent parenchymal epithelial and endothelial damage, and chronic inflammatory cell recruitment and activation, i.e., ARDS. The virus, together with RAGE ligands and a cytokine storm, enters the bloodstream, also favored by the disruption of the capillary integrity, reaching organs in which deleterious RAGE/AT1R crosstalk might be already active, and causing irreversible multiorgan damage, potentially culminating in the patient’s death. (**B**) In this scenario, the pharmacological inhibition of RAGE restores the physiological activity of the receptor, as occurs in healthy subjects, resulting in mild COVID-19 upon SARS-CoV-2 infection. The absence (in healthy subjects) or destruction (in susceptible subjects treated with RAGE inhibitors) of the deleterious RAGE/AT1R crosstalk might result in restrained lung damage and an improved immune response, avoiding the cytokine storm and favoring the production of neutralizing antibodies, and the removal of infected cells and the virus. Specific *AGER* variants might predispose patients to or protect them against COVID-19 comorbidities, and dictate the outcome of COVID-19 pathology.

Thus, RAGE and the Ang II/AT1R signaling are chronically stimulated in COVID-19 comorbidities, in which crosstalk between these two pathways has been reported to contribute to inflammation and OxS. The crosstalk between RAGE and AT1R signaling might be responsible for the inflammatory events and alveolar–capillary barrier disruption in lungs, leading to high-permeability pulmonary edema and alveolar flooding upon SARS-CoV-2 infection (Figure 3), and it might be determinant in predisposing subjects with comorbidities to develop severe COVID-19. In these subjects, SARS-CoV-2 entry and replication might lead to the additional release of RAGE ligands and further RAGE activation, reinforcing the AT1R deleterious signaling and generating a positive proinflammatory loop, culminating in ARDS, systemic cytokine storm, defective immune response, and virus escape and diffusion. As an example, the HMGB1 released from necrotic cells induces ACE2 expression in a RAGE- (but not TLR4-) dependent manner, thus sustaining SARS-CoV-2 infection [117]. RAGE/AT1R signaling crosstalk and/or AT1R-dependent transactivation of RAGE might occur in organs other than the lungs, leading to widespread inflammation and multiorgan damage (Figure 3).

## 5. *AGER* Polymorphisms with Potential Relevance in COVID-19

Several polymorphisms of the RAGE gene (*AGER*) have been reported to predispose or protect people against particular diseases. The single nucleotide polymorphism (SNP) of *AGER*, rs1800624 (-374T/A), reduces the risk of cancer and Crohn’s disease, and protects against the development of CVD in both diabetic and non-diabetic patients. Other *AGER* variants, such as rs2070600 (G82S), have been shown to favor diabetic complications and cancer. Interestingly, rs2070600 and rs2071288 *AGER* variants have been associated with an increased risk of developing COPD and ARDS or emphysema in COPD patients, respectively [118]. The *AGER* polymorphism -374T/A and the S100B polymorphism +427C/T were found to be associated with increased susceptibility to invasive aspergillosis in patients undergoing hematopoietic stem cell transplantation, when present in both transplantation counterparts or in donors only, respectively [119]. Finally, in CF patients, the *AGER* -374T/A polymorphism leads to the upregulation of RAGE expression and contributes to high IgE levels [120], and the *AGER* promoter variant, -429T/C, is associated with more severe lung disease and increased RAGE expression in vitro [121]. Thus, it is possible that different *AGER* variants might differentially predispose patients to COVID-19 comorbidities and dictate the outcome of COVID-19 pathology.

## 6. Concluding Remarks and Perspectives

In the above reported scenario, the disruption of RAGE/AT1R crosstalk in COVID-19 patients using specific RAGE inhibitors, rather than RAS inhibitors, might represent a powerful therapeutic approach with the advantage of avoiding compromising the physiological role of RAS in the maintenance of body homeostasis (Figure 3). This is because RAGE physiological expression is extremely low or absent in most tissues, and the use of RAGE inhibitors would almost selectively affect those organs in which RAGE is overexpressed and/or hyperstimulated by its ligands.

Several molecules have been identified for their efficacy as RAGE inhibitors [122]. The soluble non-transducing forms of RAGE, sRAGE and esRAGE, and synthetic fragments of the receptor represent endogenous RAGE antagonists that are able to restrain the activity of the membrane-bound receptor by binding its excess ligands [123]. However, the association of sRAGE plasma levels with the severity of COVID-19 is controversial. Although a study reported that asymptomatic COVID-19 patients showed higher serum levels of sRAGE than patients with lung involvement [79], others found that significantly higher plasma levels of sRAGE characterized COVID-19-associated ARDS compared with non-COVID-19-associated ARDS, and that plasma levels of sRAGE were associated with disease severity, the need for mechanical ventilation, and mortality in COVID-19 [124,125].

The small molecules FPS-ZM1 and TTP488 (azeliragon) have demonstrated satisfactory results in terms of RAGE inhibition, antiinflammatory effects, and safety in several experimental models of diseases, with TTP488 being investigated in clinical trials [123]. Small interfering RNAs or single-stranded DNA oligonucleotides (aptamers) targeting RAGE are useful in inhibiting RAGE expression and RAGE activity, respectively, and have been used in several models of diseases, including pulmonary arterial hypertension, renal disease, and diabetes [109]. The mutant RAGE peptide S391A-RAGE362-404 inhibited RAGE transactivation with AT1R, thus attenuating Ang II-dependent inflammation and atherogenesis in an animal model of atherosclerosis [126].

Interventions aiming to inhibit or reduce the levels of RAGE ligands also translate into the inhibition of RAGE activity. Pentamidine, a small molecule that is able to bind and inhibit S100B, reduced neuroinflammation and S100B levels in an experimental model of multiple sclerosis [127]. Arundic acid, another S100B inhibitor, prevented astrocytic activation, enhanced neuronal survival, and reversed neurological deficits and tissue damage following intracerebral hemorrhage in rats [128]. Monoclonal antibodies, peptide inhibitors, RNA interference (RNAi), ARBs, and various chemical compounds (e.g., ethyl pyruvate) have been used to inhibit the expression and release of HMGB1 in preclinical studies [129]. Interestingly, the licorice-derived extract and HMGB1 inhibitor, glycyrrhizin emerged as the most active compound among several antiviral agents in inhibiting coronavirus replication [130].

Lifestyle changes are an important means to prevent the hyperactivation of RAGE and to reduce the comorbidities associated with severe COVID-19. A reduced consumption of AGE-rich foods (red meat, animal fat, cheese, etc.), changes in cooking methods (reducing the cooking duration and temperature), and the interruption of cigarette smoking decrease the levels of AGEs in the serum [53,131].

The non-enzymatic AGE cross-link breaker alagebrium (3-phenacyl-4,5-dimethylthiazolium chloride, ALT-711), which reached phase III clinical trials, showed positive effects on cardiovascular hypertrophy, diabetes, hypertension, and vascular sclerotic pathologies. Aminoguanidine (pimagedine), a scavenger of reactive carbonyl groups (especially dicarbonyl compounds), prevents AGE formation and reduces the accumulation of exogenous (food-derived) AGEs, although clinical trials of aminoguanidine to prevent the progression of diabetic nephropathy failed due to safety concerns and a lack of efficacy [132].

Thanks to the autofluorescence typical of some AGEs, the measurement of the skin AGE content using a simple non-invasive method based on a fluorescence spectrometer is possible. The skin AGE content represents a reliable indirect measurement of the AGEs accumulated in other tissues. Skin autofluorescence was found to be significantly associated with arterial stiffness in elderly people [133]. Thus, measurement of the skin AGE levels in COVID-19 patients could be performed to evaluate the existence of a correlation between the AGE content and unfavorable outcomes of the pathology. If this is the case, skin AGE measurements might be useful to identify people more at risk of developing severe COVID-19, representing a noteworthy advantage compared to members of the RAS system, which cannot be evaluated without an invasive approach.

Current therapy for COVID-19 includes traditional prevention with vaccines [134], the use of inhibitors of the coronavirus RNA-dependent RNA polymerase (such as remdesivir) [135], and passive immunity approaches using convalescent plasma from recovered patients [136] or using neutralizing antiviral monoclonal antibodies (such as casirivimab/imdevimab, which are recombinant human monoclonal antibodies against nonoverlapping epitopes of the receptor-binding domain of the viral S protein) [137]. Although interesting, little is known about the impact exerted on RAGE signaling by the current therapeutic strategies against COVID-19. Chloroquine/hydroxychloroquine compounds exert antiinflammatory effects and have been used in China and South Korea to treat COVID-19 infections [138]. Chloroquine was shown to prevent the spread of coronavirus by interfering with ACE2 glycosylation [139]. Interestingly, chloroquine decreases HMGB1 secretion from activated immune cells, interferes with HMGB1-mediated lysosomal leakage, preventing the activation of intracellular proinflammatory receptors, and reduces NET formation in an experimental model of acute pancreatitis [11]. However, caution should be taken on the use of these autophagy-inhibiting drugs in consideration of their toxic side effects, including NF-E2-related factor 2 (Nrf2)-dependent reductive stress [140].

Based on the above-reported considerations, in-depth investigations should be performed on the inhibition of RAGE signaling as a potential therapeutic approach, and particular attention should be paid to the evaluation of the role of *AGER* polymorphisms in the context of SARS-CoV infections in order to anticipate and prevent severe COVID pathologies. Furthermore, RAGE and RAGE ligands are candidates as useful biomarkers of the severity of lung and organ damage in SARS-CoV infections.

## Data Availability

Not applicable.

## Appendix A. SARS-CoV-2 Profile

Coronaviruses belong to the subfamily Coronavirinae, family Coronaviridae, order Nidovirales. The Coronavirinae subfamily includes four groups, α, β, γ, and δ. The severe acute respiratory syndrome coronavirus 2 (SARS-CoV-2) is a betacoronavirus, identified for the first time in Wuhan, China, in December 2019. The genome of CoVs is a single-stranded positive-sense RNA (+ssRNA) of approximately 30 kb in length and with at least six open reading frames. It encodes four main structural proteins, spike (S), envelope (E), membrane (M), and nucleocapsid (N), together with substructural proteins involved in virus replication [1]. SARS-CoV-2 shares ~80% and ~50% of the genome with SARS-CoV and MERS-CoV, respectively. Its high (~95%) protein sequence homology with SARS-CoV translates into a very similar mechanism of infection: homotrimers of the S protein generate spikes at the virus surface and bind to the ACE2 transmembrane receptor, which is widely expressed in cells of the trachea, bronchi, and lung alveoli (epithelial cells and macrophages) [9]. Following SARS-CoV infection, the decrease in ACE2 amounts leads to disruption of the physiological function of the RAS, culminating in uncontrolled inflammation and ARDS. ACE2 is also expressed in the endothelial cells of blood vessels, intestinal mucosal cells, kidney epithelial tubular cells, nervous cells, endocrine cells, and immune cells, predisposing patients to SARS-CoV-2-induced multiorgan damage.

## Appendix B. The Renin Angiotensin System

Although classically involved in blood pressure regulation and body electrolyte balance, the renin angiotensin system (RAS) exerts multiple paracrine/autocrine effects in various tissues affecting organ physiology and homeostasis. In the complex RAS cascade, renin, an enzyme produced by the juxtaglomerular cells, converts the inactive substrate, angiotensinogen, into the active peptide angiotensin (Ang) I-(1–10). Ang I is further processed by the angiotensin-converting enzyme (ACE), a membrane-bound exopeptidase, to release the vasoactive Ang II-(1–8), which can bind Ang II type 1 (AT1R) or type 2 (AT2R) receptors. Activation of AT1R by Ang II translates into NF-κB-mediated inflammation, sodium retention, vasoconstriction, OxS, fibrosis, and cellular growth. AT2R exerts an inhibitory effect on the cellular responses activated by AT1R. Similarly, the activity of ACE is counterbalanced by its homolog, ACE2, the action of which is the conversion of Ang II into Ang-(1–7), which binds Mas receptor (MasR) and antagonizes the effects of Ang II/AT1R [9]. Thus, the RAS includes different angiotensin peptides that bind distinct receptor subtypes, leading to opposite signaling cascades, with Ang II/AT1R pairs sustaining deleterious effects in several organs.

## References

[1] Chen Y., Liu Q., Guo D. (2020). Emerging coronaviruses: Genome structure, replication, and pathogenesis. J. Med. Virol..

[2] Van Oosterhout C., Hall N., Ly H., Tyler K.M. (2021). COVID-19 evolution during the pandemic—Implications of new SARS-CoV-2 variants on disease control and public health policies. Virulence.

[3] He X., Cheng X., Feng X., Wan H., Chen S., Xiong M. (2021). Clinical symptom differences between mild and severe COVID-19 patients in China: A meta-analysis. Front. Public Health.

[4] Sanyaolu A., Okorie C., Marinkovic A., Patidar R., Younis K., Desai P., Hosein Z., Padda I., Mangat J., Altaf M. (2020). Comorbidity and its impact on patients with COVID-19. SN Compr. Clin. Med..

[5] Sorci G., Riuzzi F., Giambanco I., Donato R. (2013). RAGE in tissue homeostasis, repair and regeneration. Biochim. Biophys. Acta.

[6] Statista Most Common Comorbidities Observed in Coronavirus (COVID-19) Deceased Patients in Italy as of 22 July 2020. https://www.statista.com/statistics/1110949/common-comorbidities-in-covid-19-deceased-patients-in-italy/.

[7] New York State. Department of Health Top 10 Comorbidities by Age Group. https://covid19tracker.health.ny.gov/views/NYS-COVID19-Tracker/NYSDOHCOVID-19Tracker-Fatalities?%3Aembed=yes&%3Atoolbar=no.

[8] Surendra H., Elyazar I.R., Djaafara B.A., Ekawati L.L., Saraswati K., Adrian V., Widyastuti, Oktavia D., Salama N., Lina R.N. (2021). Clinical characteristics and mortality associated with COVID-19 in Jakarta, Indonesia: A hospital-based retrospective cohort study. Lancet Reg. Health West Pac..

[9] Gheblawi M., Wang K., Viveiros A., Nguyen Q., Zhong J.C., Turner A.J., Raizada M.K., Grant M.B., Oudit G.Y. (2020). Angiotensin-converting enzyme 2: SARS-CoV-2 receptor and regulator of the renin-angiotensin system: Celebrating the 20th Anniversary of the discovery of ACE2. Circ. Res..

[10] Riuzzi F., Sorci G., Sagheddu R., Chiappalupi S., Salvadori L., Donato R. (2018). RAGE in the pathophysiology of skeletal muscle. J. Cachexia Sarcopenia Muscle.

[11] Andersson W., Ottestad W., Tracey K.J. (2020). Extracellular HMGB1: A therapeutic target in severe pulmonary inflammation including COVID-19?. Mol. Med..

[12] Yang H., Hreggvidsdottir H.S., Palmblad K., Wang H., Ochani M., Li J., Lu B., Chavan S., Rosas-Ballina M., Al-Abed Y. (2010). A critical cysteine is required for HMGB1 binding to toll-like receptor 4 and activation of macrophage cytokine release. Proc. Natl. Acad. Sci. USA.

[13] Jin Y., Ji W., Yang H., Chen S., Zhang W., Duan G. (2020). Endothelial activation and dysfunction in COVID-19: From basic mechanisms to potential therapeutic approaches. Signal. Transduct. Target Ther..

[14] Kierdorf K., Fritz G. (2013). RAGE regulation and signaling in inflammation and beyond. J. Leukoc. Biol..

[15] Tay M.Z., Poh C.M., Rénia L., Macary P.A., Ng L.F.P. (2020). The trinity of COVID-19: Immunity, inflammation and intervention. Nat. Rev. Immunol..

[16] Park S., Yoon S.J., Tae H.J., Shim C.Y. (2011). RAGE and cardiovascular disease. Front. Biosci..

[17] Egaña-Gorroño L., López-Díez R., Yepuri G., Ramirez L.S., Reverdatto S., Gugger P.F., Shekhtman A., Ramasamy R., Schmidt A.M. (2020). Receptor for Advanced Glycation End products (RAGE) and mechanisms and therapeutic opportunities in diabetes and cardiovascular disease: Insights from human subjects and animal models. Front. Cardiovasc. Med..

[18] Bettiga A., Fiorio F., di Marco F., Trevisani F., Romani A., Porrini E., Salonia A., Montorsi F., Vago R. (2019). The modern western diet rich in Advanced Glycation End-products (AGEs): An overview of its impact on obesity and early progression of renal pathology. Nutrients.

[19] Prasad K., Mishra M. (2017). Do advanced glycation end products and its receptor play a role in pathophysiology of hypertension?. Int. J. Angiol..

[20] Schmidt A.M., Hasu M., Popov D., Zhang J.H., Chen J., Yan S.D., Brett J., Cao R., Kuwabara K., Costache G. (1994). Receptor for advanced glycation end products (AGEs) has a central role in vessel wall interactions and gene activation in response to circulating AGE proteins. Proc. Natl. Acad. Sci. USA.

[21] Goldin A., Beckman J.A., Schmidt A.M., Creager M.A. (2006). Advanced glycation end products: Sparking the development of diabetic vascular injury. Circulation.

[22] Quehenberger P., Bierhaus A., Fasching P., Muellner C., Klevesath M., Hong M., Stier G., Sattler M., Schleicher E., Speiser W. (2000). Endothelin 1 transcription is controlled by nuclear factor-kappaB in AGE-stimulated cultured endothelial cells. Diabetes.

[23] González-Quevedo A., García S.G., Concepción O.F., Freixas R.S., Sotolongo L.Q., Menéndez M.C., Sánchez M.P., Almirall I.F., Carriera R.F., Díaz Z.H. (2011). Increased serum S-100B and neuron specific enolase—Potential markers of early nervous system involvement in essential hypertension. Clin. Biochem..

[24] Rafikov R., Nair V., Sinari S., Babu H., Sullivan J.C., Yuan J.X., Desai A.A., Rafikova O. (2019). Gender difference in damage-mediated signaling contributes to pulmonary arterial hypertension. Antioxid. Redox. Signal..

[25] Biscetti F., Rando M.M., Nardella E., Cecchini A.L., Pecorini G., Landolfi R., Flex A. (2019). High mobility group box-1 and diabetes mellitus complications: State of the art and future perspectives. Int. J. Mol. Sci..

[26] Kraakman M.J., Lee M.K., Al-Sharea A., Dragoljevic D., Barrett T.J., Montenont E., Basu D., Heywood S., Kammoun H.L., Flynn M. (2017). Neutrophil-derived S100 calcium-binding proteins A8/A9 promote reticulated thrombocytosis and atherogenesis in diabetes. J. Clin. Investig..

[27] Tam X.H., Shiu S.W., Leng L., Bucala R., Betteridge D.J., Tan K.C. (2011). Enhanced expression of receptor for advanced glycation end-products is associated with low circulating soluble isoforms of the receptor in Type 2 diabetes. Clin. Sci..

[28] Arabi Y.M., Dehbi M., Rishu A.H., Baturcam E., Kahoul S.H., Brits R.J., Naidu B., Bouchama A. (2011). sRAGE in diabetic and non-diabetic critically ill patients: Effects of intensive insulin therapy. Crit. Care.

[29] Prasad K. (2021). AGE-RAGE stress and coronary artery disease. Int. J. Angiol..

[30] Nin J.W., Jorsal A., Ferreira I., Schalkwijk C.G., Prins M.H., Parving H.H., Tarnow L., Rossing P., Stehouwer C.D. (2010). Higher plasma soluble Receptor for Advanced Glycation End Products (sRAGE) levels are associated with incident cardiovascular disease and all-cause mortality in type 1 diabetes: A 12-year follow-up study. Diabetes.

[31] Fujisawa K., Katakami N., Kaneto H., Naka T., Takahara M., Sakamoto F., Irie Y., Miyashita K., Kubo F., Yasuda T. (2013). Circulating soluble RAGE as a predictive biomarker of cardiovascular event risk in patients with type 2 diabetes. Atherosclerosis.

[32] Colhoun H.M., Betteridge D.J., Durrington P., Hitman G., Neil A., Livingstone S., Charlton-Menys V., Bao W., Demicco D.A., Preston G.M. (2011). Total soluble and endogenous secretory receptor for advanced glycation end products as predictive biomarkers of coronary heart disease risk in patients with type 2 diabetes: An analysis from the CARDS trial. Diabetes.

[33] Cai X.Y., Lu L., Wang Y.N., Jin C., Zhang R.Y., Zhang Q., Chen Q.J., Shen W.F. (2011). Association of increased S100B, S100A6 and S100P in serum levels with acute coronary syndrome and also with the severity of myocardial infarction in cardiac tissue of rat models with ischemia-reperfusion injury. Atherosclerosis.

[34] Wang X., Xu T., Mungun D., Zhou C., Zha Z., Lu M., Fen C., Guo Y. (2019). The relationship between plasma soluble receptor for advanced glycation end products and coronary artery disease. Dis. Markers.

[35] Yan X.X., Lu L., Peng W.H., Wang L.J., Zhang Q., Zhang R.Y., Chen Q.J., Shen W.F. (2009). Increased serum HMGB1 level is associated with coronary artery disease in nondiabetic and type 2 diabetic patients. Atherosclerosis.

[36] Kosmopoulos M., Drekolias D., Zavras P.D., Piperi C., Papavassiliou A.G. (2019). Impact of advanced glycation end products (AGEs) signaling in coronary artery disease. Biochim. Biophys. Acta Mol. Basis Dis..

[37] Zhang X., Cheng M., Tong F., Su X. (2019). Association between RAGE variants and the susceptibility to atherosclerotic lesions in Chinese Han population. Exp. Ther. Med..

[38] Prasad K. (2020). AGE-RAGE stress in the pathophysiology of atrial fibrillation and its treatment. Int. J. Angiol..

[39] Xu Q., Bo L., Hu J., Geng J., Chen Y., Li X., Chen F., Song J. (2018). High mobility group box 1 was associated with thrombosis in patients with atrial fibrillation. Medicine.

[40] Mallipattu S.K., Uribarri J. (2014). Advanced glycation end product accumulation: A new enemy to target in chronic kidney disease?. Curr. Opin. Nephrol. Hypertens..

[41] Zhao Z., Hu Z., Zeng R., Yao Y. (2020). HMGB1 in kidney diseases. Life Sci..

[42] Cheng M., Liu H., Zhang D., Liu Y., Wang C., Liu F., Chen J. (2015). HMGB1 enhances the AGE-induced expression of CTGF and TGF-β via RAGE-dependent signaling in renal tubular epithelial cells. Am. J. Nephrol..

[43] Gugliucci A., Menini T. (2014). The axis AGE-RAGE-soluble RAGE and oxidative stress in chronic kidney disease. Adv. Exp. Med. Biol..

[44] Teissier T., Quersin V., Gnemmi V., Daroux M., Howsam M., Delguste F., Lemoine C., Fradin C., Schmidt A.M., Cauffiez C. (2019). Knockout of receptor for advanced glycation end-products attenuates age-related renal lesions. Aging Cell.

[45] Bruchfeld A., Qureshi A.R., Lindholm B., Barany P., Yang L., Stenvinkel P., Tracey K.J. (2008). High mobility group box protein-1 correlates with renal function in chronic kidney disease (CKD). Mol. Med..

[46] Southern L., Williams J., Esiri M.M. (2007). Immunohistochemical study of N-ε-carboxymethyl lysine (CML) in human brain: Relation to vascular dementia. BMC Neurol..

[47] Choei H., Sasaki N., Takeuchi M., Yoshida T., Ukai W., Yamagishi S., Kikuchi S., Saito T. (2004). Glyceraldehyde-derived advanced glycation end products in Alzheimer’s disease. ActaNeuropathol..

[48] Sato T., Shimogaito N., Wu X., Kikuchi S., Yamagishi S., Takeuchi M. (2006). Toxic advanced glycation end products (TAGE) theory in Alzheimer’s disease. Am. J. Alzheimers Dis. Other Dement..

[49] Shepherd C.E., Goyette J., Utter V., Rahimi F., Yang Z., Geczy C.L., Halliday G.M. (2006). Inflammatory S100A9 and S100A12 proteins in Alzheimer’s disease. Neurobiol. Aging.

[50] Cai Z., Liu N., Wang C., Qin B., Zhou Y., Xiao M., Chang L., Yan L.J., Zhao B. (2016). Role of RAGE in Alzheimer’s disease. Cell. Mol. Neurobiol..

[51] Emanuele E., D’Angelo A., Tomaino C., Binetti G., Ghidoni R., Politi P., Bernardi L., Maletta R., Bruni A.C., Geroldi D. (2005). Circulating levels of soluble receptor for advanced glycation end products in Alzheimer disease and vascular dementia. Arch. Neurol..

[52] Tang S.C., Yang K.C., Hu C.J., Chiou H.Y., Wu C.C., Jeng J.S. (2017). Elevated plasma level of soluble form of RAGE in ischemic stroke patients with dementia. Neuromol. Med..

[53] Robinson A.B., Stogsdill J.A., Lewis J.B., Wood T.T., Reynolds P.R. (2012). RAGE and tobacco smoke: Insights into modeling chronic obstructive pulmonary disease. Front. Physiol..

[54] Sukkar M.B., Wood L.G., Tooze M., Simpson J.L., McDonald V.M., Gibson P.G., Wark P.A. (2012). Soluble RAGE is deficient in neutrophilic asthma and COPD. Eur. Respir. J..

[55] Smith D.J., Yerkovich S.T., Towers M.A., Carroll M.L., Thomas R., Upham J.W. (2011). Reduced soluble receptor for advanced glycation end-products in COPD. Eur. Respir. J..

[56] Ramasamy R., Schmidt A.M. (2012). Receptor for ad-vanced glycation end products (RAGE) and implications for the pathophysiology of heart failure. Curr. Heart Fail. Rep..

[57] Koyama Y., Takeishi Y., Arimoto T., Niizeki T., Shishido T., Takahashi H., Nozaki N., Hirono O., Tsunoda Y., Nitobe J. (2007). High serum level of pentosidine, an advanced glycation end product (AGE), is a risk factor of patients with heart failure. J. Card. Fail..

[58] Wang L.J., Lu L., Zhang F.R., Chen Q.J., De Caterina R., Shen W.F. (2011). Increased serum high-mobility group box-1 and cleaved receptor for advanced glycation endproducts levels and decreased endogenous secretory receptor for advanced glycation endproducts levels in diabetic and non-diabetic patients with heart failure. Eur. J. Heart Fail..

[59] Song F., Hurtado del Pozo C., Rosario R., Zou Y.S., Ananthakrishnan R., Xu X., Patel P.R., Benoit V.M., Yan S.F., Li H. (2014). RAGE regulates the metabolic and inflammatory response to high-fat feeding in mice. Diabetes.

[60] Zhang J., Zhang L., Zhang S., Yu Q., Xiong F., Huang K., Wang C.Y., Yang P. (2017). HMGB1, an innate alarmin, plays a critical role in chronic inflammation of adipose tissue in obesity. Mol. Cell. Endocrinol..

[61] Riuzzi F., Chiappalupi S., Arcuri C., Giambanco I., Sorci G., Donato R. (2020). S100 proteins in obesity: Liaisons dangereuses. Cell. Mol. Life Sci..

[62] Boyer F., Vidot J.B., Dubourg A.G., Rondeau P., Essop M.F., Bourdon E. (2015). Oxidative stress and adipocyte biology: Focus on the role of AGEs. Oxid. Med. Cell. Longev..

[63] Davis K.E., Prasad C., Vijayagopal P., Juma S., Imrhan V. (2014). Serum soluble receptor for advanced glycation end products correlates inversely with measures of adiposity in young adults. Nutr. Res..

[64] Gaens K.H., Goossens G.H., Niessen P.M., van Greevenbroek M.M., van der Kallen C.J., Niessen H.W., Rensen S.S., Buurman W.A., Greve J.W., Blaak E.E. (2014). Nε-(carboxymethyl)lysine-receptor for advanced glycation end product axis is a key modulator of obesity-induced dysregulation of adipokine expression and insulin resistance. Arterioscler. Thromb. Vasc. Biol..

[65] Sandooja R., Vura N.V.R.K., Morocco M. (2020). Heightened ACE activity and unfavorable consequences in COVID-19 diabetic subjects. Int. J. Endocrinol..

[66] Nielsen T.B., Pantapalangkoor P., Yan J., Luna B.M., Dekitani K., Bruhn K., Tan B., Junus J., Bonomo R.A., Schmidt A.M. (2017). Diabetes exacerbates infection via hyperinflammation by signaling through TLR4 and RAGE. mBio.

[67] Sanchis-Gomar F., Lavie C.J., Mehra M.R., Henry B.M., Lippi G. (2020). Obesity and outcomes in COVID-19: When an epidemic and pandemic collide. Mayo Clin. Proc..

[68] Smith A.G., Sheridan P.A., Harp J.B., Beck M.A. (2007). Diet-induced obese mice have increased mortality and altered immune responses when infected with influenza virus. J. Nutr..

[69] Yan F., Huang F., Xu J., Yang P., Qin Y., Lv J., Zhang S., Ye L., Gong M., Liu Z. (2020). Antihypertensive drugs are associated with reduced fatal outcomes and improved clinical characteristics in elderly COVID-19 patients. Cell Discov..

[70] Semenzato L., Botton J., Drouin J., Baricault B., Vabre C., Cuenot F., Penso L., Herlemont P., Sbidian E., Weill A. (2021). Antihypertensive drugs and COVID-19 risk: A cohort study of 2 million hypertensive patients. Hypertension.

[71] Oczypok E.A., Perkins T.N., Oury T.D. (2017). All the “RAGE” in lung disease: The receptor for advanced glycation endproducts (RAGE) is a major mediator of pulmonary inflammatory responses. Paediatr. Respir. Rev..

[72] Jabaudon M., Blondonnet R., Pereira B., Cartin-Ceba R., Lichtenstern C., Mauri T., Determann R.M., Drabek T., Hubmayr R.D., Gajic O. (2018). Plasma sRAGE is independently associated with increased mortality in ARDS: A meta-analysis of individual patient data. Intensive Care Med..

[73] Li Y., Wu R., Tian Y., Yu M., Tang Y., Cheng H., Tian Z. (2015). RAGE/NF-κB signaling mediates lipopolysaccharide induced acute lung injury in neonate rat model. Int. J. Clin. Exp. Med..

[74] Van Zoelen M.A., van der Sluijs K.F., Achouiti A., Florquin S., Braun-Pater J.M., Yang H., Nawroth P.P., Tracey K.J., Bierhaus A., van der Poll T. (2009). Receptor for advanced glycation end products is detrimental during influenza A virus pneumonia. Virology.

[75] Tang Z., Zang N., Fu Y., Ye Z., Chen S., Mo S., Ren L., Liu E. (2018). HMGB1 mediates HAdV-7 infection-induced pulmonary inflammation in mice. Biochem. Biophys. Res. Commun..

[76] Vrillon A., Mhanna E., Aveneau C., Lebozec M., Grosset L., Nankam D., Albuquerque F., Feroldi R.R., Maakaroun B., Pissareva I. (2021). COVID-19 in adults with dementia: Clinical features and risk factors of mortality-a clinical cohort study on 125 patients. Alzheimers Res. Ther..

[77] Wang Q., Davis P.B., Gurney M.E., Xu R. (2021). COVID-19 and dementia: Analyses of risk, disparity, and outcomes from electronic health records in the US. Alzheimers Dement..

[78] Kanberg N., Ashton N.J., Andersson L.M., Yilmaz A., Lindh M., Nilsson S., Price R.W., Blennow K., Zetterberg H., Gisslén M. (2020). Neurochemical evidence of astrocytic and neuronal injury commonly found in COVID-19. Neurology.

[79] Kehribar D.Y., Cihangiroglu M., Sehmen E., Avci B., Capraz A., Bilgin A.Y., Gunaydin C., Ozgen M. (2021). The receptor for advanced glycation end product (RAGE) pathway in COVID-19. Biomarkers.

[80] Zeng H.L., Chen D., Yan J., Yang Q., Han Q.Q., Li S.S., Cheng L. (2020). Proteomic characteristics of bronchoalveolar lavage fluid in critical COVID-19 patients. FEBS J..

[81] Aceti A., Margarucci L.M., Scaramucci E., Orsini M., Salerno G., di Sante G., Gianfranceschi G., di Liddo R., Valeriani F., Ria F. (2020). Serum S100B protein as a marker of severity in Covid-19 patients. Sci. Rep..

[82] Silvin A., Chapuis N., Dunsmore G., Goubet A.G., Dubuisson A., Derosa L., Almire C., Hénon C., Kosmider O., Droin N. (2020). Elevated calprotectin and abnormal myeloid cell subsets discriminate severe from mild COVID-19. Cell.

[83] Chen L., Long X., Xu Q., Tan J., Wang G., Cao Y., Wei J., Luo H., Zhu H., Huang L. (2020). Elevated serum levels of S100A8/A9 and HMGB1 at hospital admission are correlated with inferior clinical outcomes in COVID-19 patients. Cell. Mol. Immunol..

[84] Krishnamoorthy P., Raj A.S., Roy S., Kumar N.S., Kumar H. (2021). Comparative transcriptome analysis of SARS-CoV, MERS-CoV, and SARS-CoV-2 to identify potential pathways for drug repurposing. Comput. Biol. Med..

[85] Gavriilaki E., Anyfanti P., Gavriilaki M., Lazaridis A., Douma S., Gkaliagkousi E. (2020). Endothelial dysfunction in COVID-19: Lessons learned from Coronaviruses. Curr. Hypertens. Rep..

[86] Arcanjo A., Logullo J., Menezes C.C.B., Giangiarulo T.C.d.S.C., dos Reis M.C., de Castro G.M.M., da Silva Fontes Y., Todeschini A.R., Freire-de-Lima L., Decoté-Ricardo D. (2020). The emerging role of neutrophil extracellular traps in severe acute respiratory syndrome coronavirus 2 (COVID-19). Sci. Rep..

[87] Cicco S., Cicco G., Racanelli V., Vacca A. (2020). Neutrophil Extracellular Traps (NETs) and Damage-Associated Molecular Patterns (DAMPs): Two potential targets for Covid-19 treatment. Mediat. Inflamm..

[88] Stark K., Philippi V., Stockhausen S., Busse J., Antonelli A., Miller M., Schubert I., Hoseinpour P., Chandraratne S., von Brühl M.L. (2016). Disulfide HMGB1 derived from platelets coordinates venous thrombosis in mice. Blood.

[89] Wu H., Li R., Pei L.G., Wei Z.H., Kang L.N., Wang L., Xie J., Xuet B. (2018). Emerging role of high mobility group box-1 in thrombosis-related diseases. Cell. Physiol. Biochem..

[90] Liao Y.H., Zheng J.Q., Zheng C.M., Lu K.C., Chao Y.C. (2020). Novel molecular evidence related to COVID-19 in patients with diabetes mellitus. J. Clin. Med..

[91] Jeong J., Lee J., Lim J., Cho S., An S., Lee M., Yoon N., Seo M., Lim S., Park S. (2019). Soluble RAGE attenuates AngII-induced endothelial hyperpermeability by disrupting HMGB1-mediated crosstalk between AT1R and RAGE. Exp. Mol. Med..

[92] Cheng C.L., Tang Y., Zheng Z., Liu X., Ye Z.C., Wang C., Lou T.Q. (2012). Advanced glycation end-products activate the renin-angiotensin system through the RAGE/PI3-K signaling pathway in podocytes. Clin. Investig. Med..

[93] Pickering R.J., Tikellis C., Rosado C.J., Tsorotes D., Dimitropoulos A., Smith M., Huet O., Seeber R.M., Abhayawardana R., Johnstone E.K. (2019). Transactivation of RAGE mediates angiotensin-induced inflammation and atherogenesis. J. Clin. Investig..

[94] Zhou S., Lu H., Chen R., Tian Y., Jiang Y., Zhang S., Ni D., Su Z., Shao X. (2018). Angiotensin II enhances the acetylation and release of HMGB1 in RAW264.7 macrophage. Cell Biol. Int..

[95] Ihara Y., Egashira K., Nakano K., Ohtani K., Kubo M., Koga J., Iwai M., Horiuchi M., Gang Z., Yamagishi S. (2007). Upregulation of the ligand-RAGE pathway via the angiotensin II type I receptor is essential in the pathogenesis of diabetic atherosclerosis. J. Mol. Cell. Cardiol..

[96] Liu X.P., Pang Y.J., Zhu W.W., Zhao T.T., Zheng M., Wang Y.B., Sun Z.J., Sun S.J. (2009). Benazepril, an angiotensin-converting enzyme inhibitor, alleviates renal injury in spontaneously hypertensive rats by inhibiting advanced glycation end-product-mediated pathways. Clin. Exp. Pharmacol. Physiol..

[97] Smith J.S., Lefkowitz R.J., Rajagopal S. (2018). Biased signalling: From simple switches to allosteric microprocessors. Nat. Rev. Drug Discov..

[98] Kikuchi K., Tancharoen S., Ito T., Morimoto-Yamashita Y., Miura N., Kawahara K., Maruyama I., Murai Y., Tanaka E. (2014). Potential of the angiotensin receptor blockers (ARBs) telmisartan, irbesartan, and candesartan for inhibiting the HMGB1/RAGE axis in prevention and acute treatment of stroke. J. Mol. Sci..

[99] Nakamura K., Yamagishi S., Nakamura Y., Takenaka K., Matsui T., Jinnouchi Y., Imaizumi T. (2005). Telmisartan inhibits expression of a receptor for advanced glycation end products (RAGE) in angiotensin-II-exposed endothelial cells and decreases serum levels of soluble RAGE in patients with essential hypertension. Microvasc. Res..

[100] Hagiwara S., Iwasaka H., Hidaka S., Hasegawa A., Koga H., Noguchi T. (2009). Antagonist of the type-1 ANG II receptor prevents against LPS-induced septic shock in rats. Intensive Care Med..

[101] Fan Q., Liao J., Kobayashi M., Yamashita M., Gu L., Gohda T., Suzuki Y., Wang L.N., Horikoshi S., Tomino Y. (2004). Candesartan reduced advanced glycation end-products accumulation and diminished nitro-OxS in type 2 diabetic KK/Ta mice. Nephrol. Dial. Transplant..

[102] Fukami K., Yamagishi S., Coughlan M.T., Harcourt B.E., Kantharidis P., Thallas-Bonke V., Okuda S., Cooper M.E., Forbes J.M. (2014). Ramipril inhibits AGE-RAGE-induced matrix metalloproteinase-2 activation in experimental diabetic nephropathy. Diabetol. Metab. Syndr..

[103] Forbes J.M., Thorpe S.R., Thallas-Bonke V., Pete J., Thomas M.C., Deemer E.R., Bassal S., El-Osta A., Long D.M., Panagiotopoulos S. (2005). Modulation of soluble receptor for advanced glycation end products by angiotensin-converting enzyme-1 inhibition in diabetic nephropathy. J. Am. Soc. Nephrol..

[104] Zhang S., Liu Y., Wang X., Yang L., Li H., Wang Y., Liu M., Zhao X., Xie Y., Yang Y. (2020). SARS-CoV-2 binds platelet ACE2 to enhance thrombosis in COVID-19. J. Hematol. Oncol..

[105] Boone B.A., Murthy P., Miller-Ocuin J., Doerfler W.R., Ellis J.T., Liang X., Ross M.A., Wallace C.T., Sperry J.L., Lotze M.T. (2018). Chloroquine reduces hypercoagulability in pancreatic cancer through inhibition of neutrophil extracellular traps. BMC Cancer.

[106] Greinacher A., Thiele T., Warkentin T.E., Weisser K., Kyrle P.A., Eichinger S. (2021). Thrombotic thrombocytopenia after ChAdOx1 nCov-19 vaccination. N. Engl. J. Med..

[107] Tremblay M.E., Madore C., Bordeleau M., Tian L., Verkhratsky A. (2020). Neuropathobiology of COVID-19: The role for glia. Front. Cell. Neurosci..

[108] Anker M.S., Landmesser U., von Haehling S., Butler J., Coats A.J.S., Anker S.D. (2021). Weight loss, malnutrition, and cachexia in COVID-19: Facts and numbers. J. Cachexia Sarcopenia Muscle.

[109] Chiappalupi S., Sorci G., Vukasinovic A., Salvadori L., Sagheddu R., Coletti D., Renga G., Romani L., Donato R., Riuzzi F. (2020). Targeting RAGE prevents muscle wasting and prolongs survival in cancer cachexia. J. Cachexia Sarcopenia Muscle.

[110] Yamamoto K., Takeshita H., Rakugi H. (2020). ACE2, angiotensin 1-7 and skeletal muscle: Review in the era of COVID-19. Clin. Sci..

[111] Takeshita H., Yamamoto K., Nozato S., Takeda M., Fukada S.I., Inagaki T., Tsuchimochi H., Shirai M., Nozato Y., Fujimoto T. (2018). Angiotensin-converting enzyme 2 deficiency accelerates and angiotensin 1-7 restores age-related muscle weakness in mice. J. Cachexia Sarcopenia Muscle.

[112] Sartiani L., Spinelli V., Laurino A., Blescia S., Raimondi L., Cerbai E., Mugelli A. (2015). Pharmacological perspectives in sarcopenia: A potential role for renin-angiotensin system blockers?. Clin. Cases Miner. Bone Metab..

[113] Anker S.D., Negassa A., Coats A.J., Afzal R., Poole-Wilson P.A., Cohn J.N., Yusuf S. (2003). Prognostic importance of weight loss in chronic heart failure and the effect of treatment with angiotensin converting-enzyme inhibitors: An observational study. Lancet.

[114] Riquelme C., Acuña M.J., Torrejón J., Rebolledo D., Cabrera D., Santos R.A., Brandan E. (2014). ACE2 is augmented in dystrophic skeletal muscle and plays a role in decreasing associated fibrosis. PLoS ONE.

[115] Sagheddu R., Chiappalupi S., Salvadori L., Riuzzi F., Donato R., Sorci G. (2018). Targeting RAGE as a potential therapeutic approach to Duchenne muscular dystrophy. Hum. Mol. Genet..

[116] Porzionato A., Emmi A., Barbon S., Boscolo-Berto R., Stecco C., Stocco E., Macchi V., de Caro R. (2020). Sympathetic activation: A potential link between comorbidities and COVID-19. FEBS J..

[117] Chen R., Huang Y., Quan J., Liu J., Wang H., Billiar T.R., Lotze M.T., Zeh H.J., Kang R., Tang D. (2020). HMGB1 as a potential biomarker and therapeutic target for severe COVID-19. Heliyon.

[118] Serveaux-Dancer M., Jabaudon M., Creveaux I., Belville C., Blondonnet R., Gross C., Constantin J.M., Blanchon L., Sapin V. (2019). Pathological implications of receptor for Advanced Glycation End-Product (AGER) gene polymorphism. Dis. Markers.

[119] Cunha C., Giovannini G., Pierini A., Bell A.S., Sorci G., Riuzzi F., Donato R., Rodrigues F., Velardi A., Aversa F. (2011). Genetically-determined hyperfunction of the S100B/RAGE axis is a risk factor for aspergillosis in stem cell transplant recipients. PLoS ONE.

[120] Iannitti R.G., Casagrande A., de Luca A., Cunha C., Sorci G., Riuzzi F., Borghi M., Galosi C., Massi-Benedetti C., Oury T.D. (2013). Hypoxia promotes danger-mediated via receptor for advanced glycation end products in cystic fibrosis. Am. J. Respir. Crit. Care Med..

[121] Beucher J., Boëlle P.Y., Busson P.F., Muselet-Charlier C., Clement A., Corvol H., French C.F. (2012). Modifier gene study investigators. AGER -429T/C is associated with an increased lung disease severity in cystic fibrosis. PLoS ONE.

[122] Chiappalupi S., Salvadori L., Vukasinovic A., Donato R., Sorci G., Riuzzi F. (2021). Targeting RAGE to prevent SARS-CoV-2-mediated multiple organ failure: Hypotheses and perspectives. Life Sci..

[123] Bongarzone S., Savickas V., Luzi F., Gee A.D. (2017). Targeting the Receptor for Advanced Glycation End products (RAGE): A medicinal chemistry perspective. J. Med. Chem..

[124] Lim A., Radujkovic A., Weigand M.A., Merle U. (2021). Soluble receptor for advanced glycation end products (sRAGE) as a biomarker of COVID-19 disease severity and indicator of the need for mechanical ventilation, ARDS and mortality. Ann. Intensive Care..

[125] Kapandji N., Yvin E., Devriese M., de Margerie-Mellon C., Moratelli G., Lemiale V., Jabaudon M., Azoulay E., Constantin J.M., Dumas G. (2021). Importance of lung epithelial injury in COVID-19 associated acute respiratory distress syndrome: Value of plasma sRAGE. Am. J. Respir. Crit. Care Med..

[126] Le Bagge S., Fotheringham A.K., Leung S.S., Forbes J.M. (2020). Targeting the receptor for advanced glycation end products (RAGE) in type 1 diabetes. Med. Res. Rev..

[127] Di Sante G., Amadio S., Sampaolese B., Clementi M.E., Valentini M., Volonté C., Casalbore P., Ria F., Michetti F. (2020). The S100B inhibitor pentamidine ameliorates clinical score and neuropathology of relapsing-remitting multiple sclerosis mouse model. Cells.

[128] Cordeiro J.L., Neves J.D., Vizuete A.F., Aristimunha D., Pedroso T.A., Sanches E.F., Gonçalves C.A., Netto C.A. (2020). Arundic acid (ONO-2506), an inhibitor of S100B protein synthesis, prevents neurological deficits and brain tissue damage following intracerebral hemorrhage in male wistar rats. Neuroscience.

[129] Musumeci D., Roviello G.N., Montesarchio D. (2014). An overview on HMGB1 inhibitors as potential therapeutic agents in HMGB1-related pathologies. Pharmacol. Ther..

[130] Cinatl J., Morgenstern B., Bauer G., Chandra P., Rabenau H., Doerr H.W. (2003). Glycyrrhizin, an active component of liquorice roots, and replication of SARS-associated coronavirus. Lancet.

[131] Uribarri J., Cai W., Sandu O., Peppa M., Goldberg T., Vlassara H. (2005). Diet-derived advanced glycation end products are major contributors to the body’s AGE pool and induce inflammation in healthy subjects. Ann. N. Y. Acad. Sci..

[132] Ramasamy R., Vannucci S.J., Yan S.S., Herold K., Yan S.F., Schmidt A.M. (2005). Advanced glycation end products and RAGE: A common thread in aging, diabetes, neurodegeneration, and inflammation. Glycobiology.

[133] Stürmer M., Šebeková K., Fazeli G., Bahner U., Stäb F., Heidland A. (2015). 25-hydroxyvitamin d and advanced glycation endproducts in healthy and hypertensive subjects: Are there interactions?. J. Ren. Nutr..

[134] Sandor A.M., Sturdivant M.S., Ting J.P.Y. (2021). Influenza virus and SARS-CoV-2 vaccines. J. Immunol..

[135] Kokic G., Hillen H.S., Tegunov D., Dienemann C., Seitz F., Schmitzova J., Farnung L., Siewert A., Höbartner C., Cramer P. (2021). Mechanism of SARS-CoV-2 polymerase stalling by remdesivir. Nat. Commun..

[136] Casadevall A., Pirofski L.A. (2020). The convalescent sera option for containing COVID-19. J. Clin. Investig..

[137] Hansen J., Baum A., Pascal K.E., Russo V., Giordano S., Wloga E., Fulton B.O., Yan Y., Koon K., Patel K. (2020). Studies in humanized mice and convalescent humans yield a SARS-CoV-2 antibody cocktail. Science.

[138] Gao J., Tian Z., Yang X. (2020). Breakthrough: Chloroquine phosphate has shown apparent efficacy in treatment of COVID-19 associated pneumonia in clinical studies. Biosci. Trends.

[139] Vincent M.J., Bergeron E., Benjannet S., Erickson B.R., Rollin P.E., Ksiazek T.G., Seidah N.G., Nichol S.T. (2005). Chloroquine is a potent inhibitor of SARS coronavirus infection and spread. Virol. J..

[140] Bellezza I., Riuzzi F., Chiappalupi S., Arcuri C., Giambanco I., Sorci G., Donato R. (2020). Reductive stress in striated muscle cells. Cell. Mol. Life Sci..

